# The Comforts and Feeding of the Wounded during Transit

**Published:** 1915-04-17

**Authors:** George Beatson

**Affiliations:** Chairman Scottish Branch, British Red Cross Society.


### THE COMFORTS AND FEEDING OF THE WOUNDED DURING TRANSIT.

### A Review of the Supply Organisation.

By Sir GEORGE BEATSON, K.C.B., Chairman Scottish Branch, British Red Cross Society.

Transport has become the serious problem of every campaign, the dominant factor in it being how to provide at the Front ammunition for the army in the field and food for the men and horses, as also how to take back from the Front all that is not required with the army, such as sick, wounded, and prisoners of war. To allow of a better understanding as to how this difficult matter of transport has been met, it is necessary briefly to outline the supply organisation in use at present in our Expeditionary Force, especially as it will be seen that it is based on a general system and that all the different departments of the army, the medical included, have to work into it and are a part of it.

### The Transport Service.

The departmental organisation for the transport and supply arrangements of our army is known as the Army Service Corps, and was formed in 1888. It has been developed with the greatest thought and care, and is now probably the best in the world, so that we are able very justly to boast that never in the history of war have troops been better fed than those of our present Expeditionary Force on the Continent of Europe. Without going minutely into detail it may be pointed out that the ordinary supply scheme of the British Army has three zones. The first one is the line of railway or of waterway from the main base to the advance base or railhead and is usually worked by trains. The second zone extends from the railhead to a point known as the refilling point, which is usually from four to five miles behind the firing line, and is carried out by motor vehicles. The third and last zone is a short one, and reaches from the refilling point to the camps of the different regiments and units of the Division or Army Corps that has to be supplied, and is accomplished by horse waggons. This zone has a fan-like radiation as the vehicles proceed from it and distribute supplies to the various commands. In this way there is a constant stream of supplies, both of munitions and of food and stores, flowing from the base to the Front, all the zones being linked up without a gap. Further, each zone has its own appropriate mode of transport, experience and thought having decided which type of vehicle is best adapted to each portion of the transit. So far the arrangement is that horse transport companies carry out the actual distribution of food, forage, fuel, and ammunition to the troops of the fighting front, it being found that horse waggons are best suited for this purpose, the work having often to be done over rough and roadless ground. For the other zones transport in the shape of the railway or motor vehicles is employed. In this way an attempt has been made to solve the pressing problem of transport in modern warfare, and it has now been made quite clear that relief can only be got by utilising motor vehicles and by following a line of strategy which will allow of the utilisation of the railway. In fact, no campaign can be waged nowadays except in close proximity to main lines of railway.

The problem of removing the sick and wounded from the front of an army is very similar to the bringing up of its supplies, only in the reverse direction, and if we recall the Scheme of Medical Arrangements in War as they exist in our British Army we at once recognise that they, too, fall into three zones—the collecting, evacuating, and distributing. The same principle underlies all of them, viz: Evacuation. There is also a close relation in these three zones of Supply and Evacuation, although reversed, for the Dressing Station of the Wounded corresponds to the Refilling Point, the Casualty Clearing Station to the Railhead, and the Base Hospital to the Main Base of Supply. So in the same way the transport arrangements from Dressing Station to Field Ambulance, and from Field Ambulance to Casualty Clearing Station are by horse waggons, just as they are for the regimental distribution of stores, and from Casualty Clearing Station to railhead and then to base by motor vehicles or by trains just as in the Supply Service, the same trains that carried the great bulk of the stores to the Front being utilised for bringing back the great mass of the wounded, thus employing the empty return waggons and trains on the principle that each department must be subordinate to the general supply scheme, and that with an army in the field there should be no waste of energy or material. It will also be noticed that in both the supply and medical arrangements the transport that belongs to the Front and that which works the base are separate and distinct and never mix, the horse waggons of the Field Ambulances never leaving their units except to hand over their wounded to the Casualty Clearing Station, and then never for more than twelve or, at the outside, twenty-four hours.

### Improving the Means of Transit.

Thus, the problem of evacuating the sick and wounded during a campaign has been based in the British Army on the plan of making use of the empty supply waggons and railway trucks of trains of the Army Medical Service Corps on their return to the base, special hospital trains carrying the ordinary sick and severely wounded only. This scheme, by which the R.A.M.C. depends upon the Supply Corps, has its disadvantages, and the officers of the latter corps are against the idea of carrying sick and wounded, giving, among other reasons.

the danger of spreading disease, a very real one in some cases. In the course of the present campaign on the Continent these objections have practically been acknowledged, and it was felt that between the Field Ambulances and the base a different arrangement was needed to ensure greater rapidity of removal and greater comfort for the wounded during transit. As far back as the month of September, 1914, the military authorities recognised that in the motor ambulance they had an improved means of transit, and one that was really a necessity, because there had been such a destruction of railway lines and bridges that trains were not available, with the result that there was great delay and suffering in connection with the evacuation of the sick and wounded. A request for a supply of motor ambulances was put before the public by the British Red Cross Society and its Scottish branch, and a most generous and hearty response followed, thus allowing the wounded to be brought practically from the firing line down to the base by these vehicles if the train service was disorganised. In addition there was a large increase in the number of permanent ambulance trains, so that when the railway was available the wounded travelled under very much better conditions than prevailed when they were carried by temporary or improvised ambulance trains.

### The Links in the Service.

It is not necessary in this first article to detail the construction of a permanent ambulance train beyond saying that it consists of special ambulance coaches fitted with tiers of cots, compartments for the medical officers, the nursing sisters, and the remaining *personnel*, a treatment room, dispensary, and kitchen. As regards the motor ambulances, the experience gained during the campaign has materially improved them, and the most approved type accommodates four patients lying, two sitting, with the attendant, or two lying and three sitting, there being a passage in the centre of the waggon to allow of the lying cases being inspected and attended to. Already some hundred motor ambulances have been dispatched to the Continent to be employed in the transit of our wounded, and they are still being provided generously by the public. They practically now do the work between the Field Ambulances close to the firing line and the Casualty Clearing Stations, and from the latter to the railhead, where they get into touch with the special ambulance trains. Of these latter there are now a considerable number on the Continent in use by the Allies, while at home no fewer than twelve permanent ambulance trains have been constructed for service in this country, and by them the sick and wounded are taken from the receiving station at Southampton to the different first-line hospitals throughout the kingdom. To bridge the gap between the Continent and Great Britain numerous hospital ships have also been constructed by the conversion of many of our large passenger steamers, and on these all the arrangements are specially adapted to ensure the greatest ease and comfort in travelling.

In addition, however, to comfortable transport it is necessary that the sick and wounded soldier should have suitable feeding and comforts during transit, and probably the best way to illustrate how admirably this has been provided for is briefly to sketch the means furnished at the different stages of his journey from the Front to the base, commencing with the Field Dressing Station.

### Field Dressing Station.

Here the means available are supporting and stimulating beverages, as warm soups, tea, and brandy, recourse being had to the medical comfort pannier for helping to furnish this improvised food, as from this pannier can be obtained arrowroot, meat extracts, condensed milk, sago, sugar, tea, brandy, etc.

### Field Ambulance.

This is what was known as the old Field Hospital, and in it are found tents sufficient to accommodate 150 wounded, with an operating tent. Of course, if possible, a building may take the place of this tented unit. Assuming that tents are used, then kitchens are dug and cooks get ready hot drinks and soups and prepare meals for the wounded. Here, again, the medical comfort pannier may have to be utilised, but "rations" will have been requisitioned from the Army Service Corps, while the wounded may have their rations of meat and biscuits in their haversacks, and these can be used. In addition to the field kitchens each of three sections of a Field Ambulance has in its equipment a cooking stove that will cook with either wood or coal for 50 persons, and as each section has two cooks, it is clear that with six cooks and with three stoves provided for 150 persons, in addition to the field kitchen, and with the bread and meat of the "rations," the bovril, and medical comforts of the medical comfort pannier, ample provision has been made for the feeding of the wounded.

### Casualty Clearing Station.

This is the more recent name for the Clearing Hospital, and it is to it the wounded are removed after their short stay in the Field Ambulance. It has accommodation nominally for 200 patients, but it may be extended so as to meet additional demands. In it for the first time in this campaign nursing sisters are employed, as it is usually placed behind shell fire and consists of a converted building. Of its staff, four are cooks and seventeen are nursing orderlies. Being what is termed a "non-dieted" hospital, it indents direct on the Army Service Corps and gets its supplies from the Advanced Supply Depôt, so that supplementary comforts of all kinds can be obtained, and, as the hospital has its full supply of panniers, there is also the medical comfort one to fall back on. Should this Casualty Clearing Station not be in a building with kitchen accommodation, then improvised kitchens, with stoves, would have to be used. Being, as it really is, a Relief Hospital into which the Field Ambulances pass their wounded, it may change its location so as to keep in touch with the Front. Consequently, it is not a stationary hospital in the true sense of the word, but the aim is always to have it as near as possible to the railhead. No matter, however, where it is placed, looking to the staff it has of doctors, nursing sisters, and trained cooks, it may be taken for granted that its inmates are provided with suitable and well-prepared food.

### Stationary Hospitals.

To these the wounded man comes either by motor ambulance or by hospital train, and in each of these the feeding arrangements have not been lost sight of. In the case of the motor ambulance, these are more or less of an improvised kind, but if the ambulance forms one of the fleet or convoy, then there usually accompanies the unit a well-furnished motor kitchen with every facility for cooking purposes. The same holds good for the ambulance train, which on the Continent is equipped for 350 cases, and at home for 100. It is provided with well-appointed berths, a trained nursing staff, food, and a kitchen with qualified cooks. Its duty is to run between the railhead and the base, but if the lines of communication are long it will probably be confined to one section of the line, and on the line will be located at suitable distances stationary hospitals. In these latter will be placed cases that may shortly recover or are too ill to stand a long journey to the base. As the stationary hospital is a " dieted " one, it can requisition on the Army Service Corps not only for the ordinary rations which form the basis of the ordinary hospital and convalescent diet, but requisition may be made for special diets, as those of milk, fish, and chicken, and for eggs or other extras. All such diets are fixed by regulation, but the medical officer can always make additions to them and can include in them any stimulants he thinks necessary.

A hospital that may make demands of such a generous but varied nature must be placed where such supplies are available, and, consequently, stationary hospitals are usually located near large or prosperous towns. In connection with the above remarks on ambulance trains, it should be observed that reference was made to the permanent ones, but in cases where temporary or improvised ambulance trains are used, that are not, as a rule, furnished with special kitchen accommodation, it is customary to have " rest stations " established along the line, and at these the trains stop for feeding the patients. In the Regular Army these are provided by the Army Service Corps, but in this country usually by the Voluntary Aid Detachments of the Territorial Medical Service.

### The General Hospitals at the Base.

In these the same dieting arrangements exist as in a stationary hospital, which is really a small general hospital, and sometimes expands into it. Both of them are " dieted " hospitals, and both have the same nursing and cooking arrangements, so that the patients may have not only ordinary diets, but the special ones of milk, fish, and chicken already mentioned above.

A hospital ship is really a floating hospital, and when on board it, whether the passage be short or long, there is the same care bestowed on its sick and wounded passengers. It is staffed by nursing sisters and by R.A.M.C. orderlies, and is a dieted hospital, the only point of difference being that the stores are put on board by the Admiralty and not by the Army Service Corps.

### The End of the Journey.

With the arrival of the soldier at the base general hospital his transit as a sick or wounded individual ceases, and there he remains until he is returned to his depôt or his regimental unit. Under certain circumstances he may be sent to a convalescent camp or home to recruit further, and so every care is taken that his journey there shall be made comfortable and devoid of risk. This duty has been undertaken, and most efficiently carried out, by the British Red Cross Society, who at the different railway termini have established what are known as Red Cross Railway Rest Stations. One has only to peruse the weekly summaries of work issued to see how both in England and in Scotland these railway rest stations have accomplished a splendid record of work. Located in rooms which have been generously provided by the railway companies free of charge, they are furnished with special equipment in the way of cooking utensils, feeding cups and saucers, kettles, and so on, and they provide warm refreshments to invalid soldiers passing through the stations, while they also serve as resting places for the sick and wounded while waiting for their trains, or for those who have arrived at the railway stations during the early hours of the morning.

### Modern Organisation.

When we read of all that our troops are enduring at the Front, it is only right and proper that we should from time to time ask ourselves if we are doing all we can to mitigate the sufferings of the sick and wounded of the campaign. A perusal of what is being done for them as detailed above justifies the reply that we believe we are, and entitles us to state that never in the history of warfare has an army been better fed or provided with a better Medical Service than in the present campaign.

There is on this occasion an absence of that want of organisation that led to the horrors of the Crimean War, when men died in such numbers that it was a question whether the whole British Army would not be wiped out in a few months. From the moment that he falls sick or wounded at the Front the British soldier in the present war is now quickly and carefully removed to the general hospital at the base, and during every stage of his transit is gently tended and suitably fed. For this great credit must be given to our Army Medical Service and to our Army Service Corps, while a word of praise is also due to the British Red Cross Society and the Order of St. John of Jerusalem, whose members have given invaluable help, and have shown that they realise that it is incumbent on them to pay their debt to their country by helping its soldiers, and in this way take a share in Imperial Defence.

